# Silicon@Carbon Composite with Bioinspired Root-Nodule Nanostructures as Anode for High-Performance Lithium-Ion Batteries

**DOI:** 10.3390/molecules30214157

**Published:** 2025-10-22

**Authors:** Yitong Sun, Lei Zhao, Ning Mi, Jiahao He, Jiantie Xu

**Affiliations:** 1 School of Materials Engineering, Longdong University, Qingyang 745000, China; ldxymining@126.com (N.M.); 2021131054@chd.edu.cn (J.H.); 2 Experimental Teaching Center of Mechanical Engineering, School of Intelligent Manufacturing, Longdong University, Qingyang 745000, China; 3 School of Physics and Optoelectronics, South China University of Technology, Guangzhou 510640, China

**Keywords:** bio-inspired structure, self-assembly, silicon@carbon composite anode, lithium–ion batteries

## Abstract

Silicon (Si) is a promising high-capacity anode material for lithium–ion batteries but faces challenges such as severe volume fluctuations during cycles and the formation of unstable solid-electrolyte interphase films on the electrode surface. To address these limitations, we developed a bioinspired Si@C composite anode through polydopamine-mediated self-assembly of aromatic polyamide nanofibers and nano–Si, followed by controlled pyrolysis at 1000 °C under N_2_. The resulting hierarchical architecture mimics the symbiotic root-nodule structure of legumes, featuring vascular bundle-like carbon frameworks and chemically bonded Si/C interfaces. The optimized composite delivers an initial capacity of 1107.0 mAh g^−1^ at 0.1 A g^−1^ and retains 580.0 mAh g^−1^ after 100 cycles with 52.4% retention. The exceptional electrochemical properties arise from the optimized architecture and surface interactions. The nature-inspired carbon network minimizes ionic transport resistance via vertically aligned porous pathways while simultaneously boosting lithium–ion adsorption capacity. Furthermore, radially aligned graphitic ribbons are generated through controlled polyamide thermal transformation that effectively mitigates electrode swelling and maintains stable interfacial layers during cycling.

## 1. Introduction

From portable electronic devices in daily life to electric vehicles that drive the green transformation in the transportation sector, and further to large-scale energy storage systems that support the absorption of renewable energy (e.g., wind energy and solar energy) and grid peak shaving, lithium–ion batteries (LIBs) serve as the core energy storage carriers. The continuous breakthrough in their electrochemical performance has always been a key research focus shared by the global scientific community and industry [1,2,3,4]. Whether it is the demand for “fast charging, long battery life, and miniaturization” of batteries in consumer electronics, the pursuit of “high energy density, long cycle life, and high safety” for electric vehicles, or the requirement for “low cost, high rate capability, and wide temperature range adaptability” in large-scale energy storage systems, all essentially rely on the iterative upgrading of the comprehensive performance of lithium–ion batteries. The key breakthrough point in this upgrading process is concentrated on the innovative research and development of core battery materials. As the core component of lithium–ion batteries for “charge storage and transfer”, the performance of anode materials directly and profoundly affects the energy density, cycle stability, rate capability, and safety performance of batteries and largely determines the comprehensive performance of the entire battery system [5,6]. Silicon has been regarded as one of the most promising anode materials for LIBs to replace the most commonly used graphite owing to its ultrahigh theoretical capacity of 3580 mAh g^−1^ [7]. However, its practical applications are severely hindered by its poor rate capability and cycling stability, resulting from poor electrical conductivity, substantial volume fluctuations, and interfacial side reactions during the cycles [8].

To address these limitations, various approaches have been systematically developed, including material nanoengineering [9], composite fabrication [10,11,12], electrolyte optimization [13], and crystallographic modification [14]. Nanoengineering of silicon (e.g., nanowires [15], nanotubes [16], and porous architectures [17]) effectively mitigates volume fluctuations during the cycles. Advanced Si@carbon (Si@C) composites incorporating highly conductive agents (e.g., graphene [18,19], carbon nanotubes [20,21], graphite [22,23], and three-dimensional (3D) carbon frameworks [24]) synergistically enhance electrical percolation networks, accommodate volume fluctuations, and prevent excessive electrolyte penetration [25,26]. The optimization of electrolytes through fluorinated additives (e.g., fluoroethylene carbonate [27]) and innovative formulations such as localized high-concentration electrolytes [13] and solid-state electrolytes [28,29] enables silicon with the formation of stable and robust solid-electrolyte interfaces (SEI) films in Si-based systems. Crystallographic modulation via defect engineering could significantly enhance lithium diffusion kinetics in Si [30]. Among these strategies, the fabrication of hierarchical Si@C composite architectures represents a technologically viable pathway toward commercialization of high-performance Si-based anodes [31,32]. For instance, Chen et al. [23] achieved cross-linking through the Maillard reaction between sodium carboxymethyl cellulose and thiourea, constructing a microscale structure that connects silicon nanoparticles and carbon additives. This further forms a Si@C composite with excellent conductive pathways. The composite not only reduces volume deformation during the charge–discharge process but also lowers the charge transfer resistance. After 500 cycles, it still maintains a capacity of 1070 mAh g^−1^.

Herein, inspired by the structure of root nodules in leguminous plants and their nitrogen-fixing mechanism [33,34], we developed a bioinspired Si@C anode material by integrating nano–silicon with aromatic polyamide nanofibers through polydopamine (PDA)-mediated self-assembly, followed by controlled pyrolysis at 1000 °C under an N_2_ atmosphere. This process yields a hierarchical architecture mimicking the root nodule symbiosis of leguminous plants, characterized by vascular bundle-like carbon frameworks, nodule-like Si@C, and chemically bonded Si/carbon interfaces. The optimized composite exhibits an initial capacity of 1107.0 mAh g^−1^ at a current density of 0.1 A g^−1^ and retains 580.0 mAh g^−1^ after 100 cycles. The performance stems from synergistic structural and interfacial engineering in which the biomimetic fibrous carbon networks reduce ionic diffusion tortuosity through 3D interconnected channels, and the biomimetic nodule-like Si@C enhances Li^+^ adsorption energy while carbon shells derived from polyamide decomposition accommodate volume fluctuations and suppress the degradation of SEI films.

## 2. Results and Discussion

**Figure 1:**
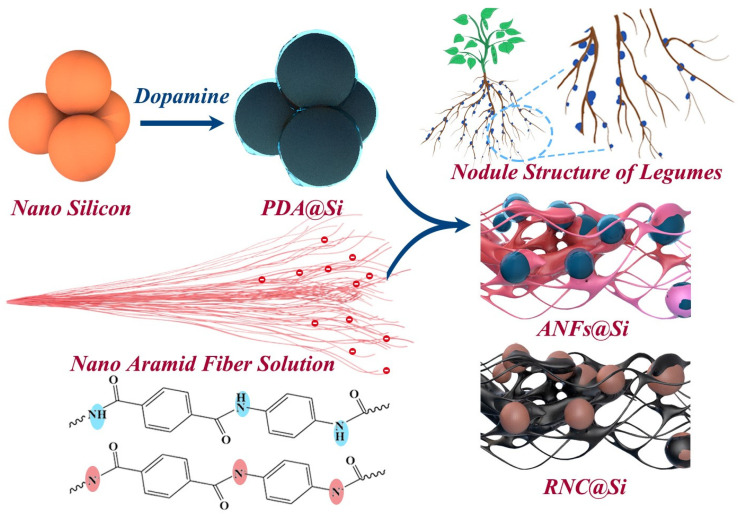
Schematic illustration for the synthesis process of bioinspired RNC@Si-X composite (the blue background on the molecular chains of nano aramid fibers emphasizes the functional groups that have not lost protons, while the rose-red background emphasizes the functional groups after losing protons).

As illustrated in Figure 1, the RNC@Si-X composites were fabricated through a step-by-step assembly strategy driven by synergistic electrostatic and hydrogen-bonding interactions among silicon nanoparticles, PDA, and ANFs. In the composites, PDA acts as a multifunctional bridge in which its catechol groups electrostatically adsorb onto hydroxyl-terminated silicon surfaces while amino/hydroxyl moieties form hydrogen bonds with ANFs. The ANFs, derived from Kevlar fibers via alkali-induced deprotonation, contribute negatively charged sulfonic groups that further enhance interfacial adhesion to PDA-modified silicon nanoparticles [35]. Under alkaline DMSO/KOH conditions, dopamine undergoes oxidative polymerization into PDA, which simultaneously cross-links silicon nanoparticles and ANFs into a 3D hybrid network. Subsequent pyrolysis converts this organic-inorganic framework into a hierarchical root-nodule-inspired architecture in which silicon nanoparticles are embedded within a conductive carbon matrix intertwined with a carbon shell. This bioinspired RNC@Si-X composite mimics legume root nodule symbiosis. The nodule-like Si@C domains serve as active Li^+^ alloying sites, while the fibrous carbon networks enhance conductivity. In addition, symbiotic pores from fibrous carbon networks enable electrolyte infiltration, and the carbon shell covering silicon particles buffers the volume fluctuations of silicon during cycling.

**Figure 2:**
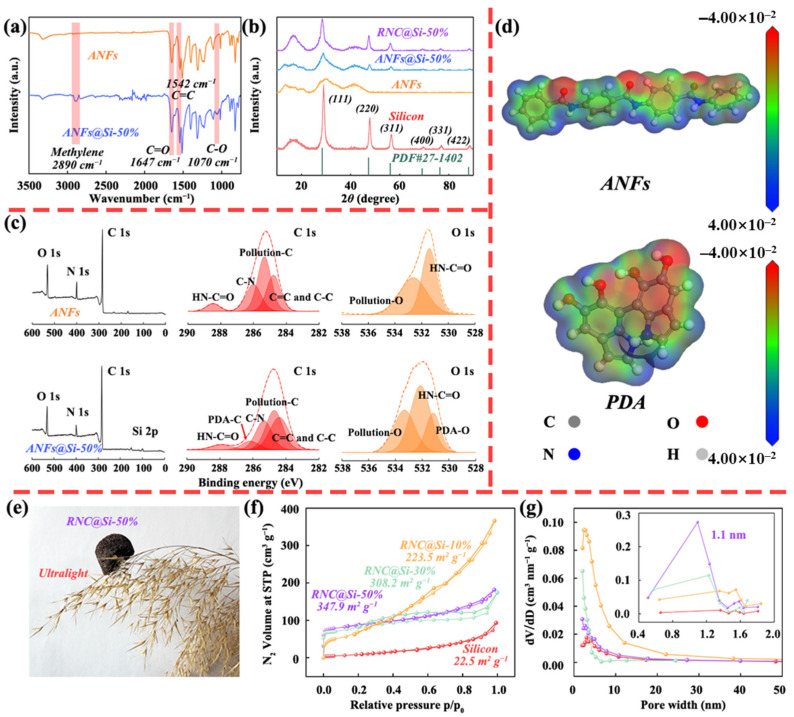
(**a**) FT-IR spectra of ANFs and ANFs@Si-50%, (**b**) XRD patterns of pristine Si, ANFs, ANFs@Si-50%, and RNC@Si-50%, (**c**) XPS spectra of ANFs and ANFs@Si-50%, (**d**) the charge distributions of ANFs and PDA surface molecule, (**e**) Digital photograph of RNC@Si-50% and (**f**) N_2_ adsorption and desorption curves and (**g**) pore size distribution curves of RNC@Si-10%, RNC@Si-30%, RNC@Si-50%, and pristine Si (The red line, yellow line, green line, and purple line represent silicon, RNC@Si-10%, RNC@Si-30%, and RNC@Si-50%, respectively).

The chemical structure of ANFs@Si-50% was investigated by FTIR. As shown in Figure 2a, characteristic peaks corresponding to both PDA and ANFs were observed in the composite. Two stretching vibration peaks of methylene at 2890 cm^−1^ and C-O functional groups at 1070 cm^−1^ confirm the presence of PDA [36]. Additional peaks at wavenumbers 1315, 1542, and 1647 cm^−1^, corresponding to the characteristic peaks of Ph-N, C=C, and C=O in ANFs [37], respectively, verify successful integration of PDA and ANFs. XRD analysis in Figure 2b was further employed to reveal the crystalline phase evolution of ANFs@Si-50%. As can be seen, the ANFs@Si-50% exhibit diffraction peaks at 28°, 47°, and 56°, respectively, indexed to the (111), (220), and (311) planes of single-crystal Si, along with a broad peak at 41° attributed to amorphous ANFs. After the pyrolysis, RNC@Si-50% retains silicon crystallinity, while the ANF-derived amorphous carbon broadens the background signal.

XPS spectra were carried out to elucidate the chemical composition and oxidation states of pristine Si, ANFs, and ANFs@Si-50%. As shown in Figure 2c, the survey confirms dominant C, N, O, and silicon elements in ANFs@Si-50%, with N and O originating from PDA and ANFs. High-resolution C 1s spectra exhibit a peak at 286.2 eV (C-N/C-O bonds in PDA), while the O 1s spectra show a hydroxyl-related peak at 531.2 eV (PDA-OH). Notably, the HN-C=O peak of the O1s spectrum in ANFs shifts from 531.4 to 532.0 eV in ANFs@Si-50%, indicating the hydrogen bonding between PDA and ANFs. Electrostatic potential simulations in Figure 2d via Gaussian calculations reveal polar distributions in both ANFs and PDA molecules. The complementary charge densities between the N-H groups of PDA and the C=O moieties of ANFs facilitate robust hydrogen-bond-driven self-assembly, theoretically validating the composite design.

The ultra-lightweight nature of RNC@Si-50% was evident from its macroscopic morphology in Figure 2e. To quantitatively evaluate specific surface area and pore structure, Brunauer–Emmett–Teller (BET) analysis was conducted on both pristine silicon and RNC@Si-X. The BET results of RNC@Si-10%, RNC@Si-30%, RNC@Si-50%, and pristine silicon are provided in Figure 2f,g. As can be seen, the RNC@Si-50% exhibits a much higher specific surface area of 347.9 m^2^ g^−1^ than pristine silicon with only 12.5 m^2^ g^−1^ and a shift to micropore dominance centering at 1.1 nm. This is mainly due to silicon nanoparticles acting as spacers that suppress carbon agglomeration and promote micropore formation during ANF pyrolysis, leading to the reduced proportion of mesopores.

**Figure 3:**
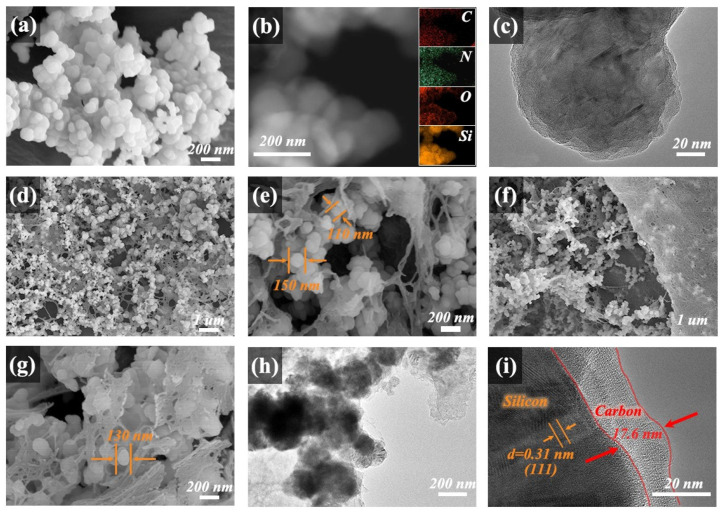
(**a**) SEM images of pristine Si; (**b**) SEM-EDS and (**c**) HRTEM of PDA@Si; the SEM images of (**d**,**e**) ANFs@Si-50% and (**f**,**g**) RNC@Si-50%; (**h**) TEM and (**i**) HRTEM images of RNC@Si-50%.

The nanostructural evolution of pristine Si, ANFs, ANFs@Si-50%, PDA@Si, and RNC@Si-50% was systematically investigated by SEM, TEM, and high-resolution TEM (HRTEM). As shown in Figure 3a, pristine silicon nanoparticles exhibit spherical morphology with diameters of 100~200 nm, while ANFs display uniform fibrous structures with diameters of <50 nm. As can be seen, the decomposition of PDAs on the surfaces of silicon was also confirmed by SEM-EDS uniform elemental mappings of additional C, N, and O in Figure 3b and the TEM image with clear and thin edges in Figure 3c. Upon composite formation in Figure 3d,e, the ANF@Si-50% displays a 3D network reminiscent of legume root nodules in which silicon nanoparticles are encapsulated within PDA coatings and interconnected by ANFs. The formation of interconnected pores by the interlaced root-nodule architecture is favorable for electrolyte penetration and ion diffusion. Owing to the direct carbonization of the thermally stable conjugated structure of ANFs, the RNC@Si-50% after pyrolysis at 1000 °C retains its root-nodule configuration and porous characteristics, as shown in Figure 3f,g. In the RNC@Si-50% composite, silicon nanoparticles are embedded within graphitic carbon shells derived from PDA, while interconnected carbon nanofibers derived from ANFs establish a continuous conductive network. This dual-carbon confinement simultaneously enhances electronic conductivity and accommodates the volume fluctuations.

Further TEM and HRTEM analysis in Figure 3h,i reveals minimal silicon nanoparticle agglomeration in RNC@Si-50% due to the restraining effect of the fibrous carbon matrix. HRTEM in Figure 3h confirms intimate contact between the carbon shell and silicon surface, with carbon nanofibers seamlessly bridging adjacent particles. HRTEM in Figure 3i further reveals lattice fringes with a d-spacing of 0.31 nm corresponding to the (111) plane of crystalline silicon (Figure S1 in Supporting Information), surrounded by amorphous carbon layers with a thickness of ~17.6 nm from PDA and ANF carbonization. These microstructural features collectively validate the successful fabrication of the biomimetic root-nodule architecture.

**Figure 4:**
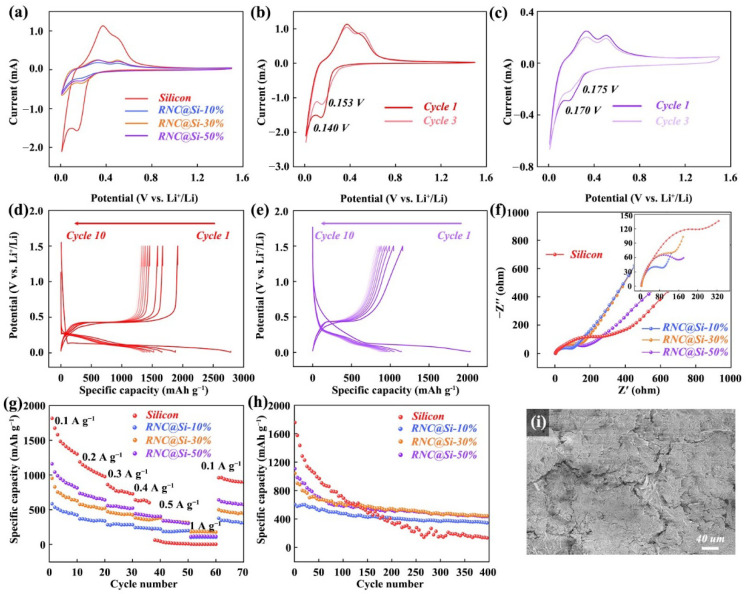
(**a**) Initial CV curves at 0.2 mV s^−1^ of pristine silicon and RNC@Si-X; CV curves at 0.2 mV s^−1^ for initial three cycles of (**b**) pristine silicon and (**c**) RNC@Si-50%; initial 10 cycles of GCD curves at 0.1 A g^−1^ of (**d**) pristine silicon and (**e**) RNC@Si-50%; (**f**) EIS spectra; (**g**) rate performance; and (**h**) cycle performance at 0.1 A g^−1^ of pristine silicon and RNC@Si-X. (**i**) The SEM images of cycled RNC@Si-50% electrode after 300 cycle numbers.

The lithium storage properties of pristine silicon and RNC@Si-X (X = 10%, 30%, and 50%) were systematically evaluated through CV, GCD, and EIS in Figure 4. The initial cycle cathodic shoulder near 0.8 V is attributed to the formation of an irreversible SEI film through electrolyte decomposition, accounting for initial capacity loss. CV curves analysis of pristine silicon and the RNC@Si-50% composite (Figure 4a–c) reveals that the composite exhibits more stabilized redox peaks. Over three consecutive cycles, the composite shows a much smaller peak potential shift (ΔEp = 0.005 V) compared with pristine silicon (ΔEp = 0.013 V). Specifically, the two observed oxidation peaks are attributed to the typical delithiation process of silicon alloys, while the emerging reduction peak corresponds to the alloying reaction of amorphous silicon. This phenomenon indicates that the unique root-nodule architecture effectively restricts the contact between silicon and the electrolyte, thereby suppressing the occurrence of parasitic reactions [21,32]. The redox characteristics of silicon and RNC@Si-50% during the 1st and 200th cycles can be more directly observed from the dQ/dV curves (Figure S2 in Supporting Information). It can be seen that silicon exhibits almost no redox characteristics after 200 cycles, indicating that silicon no longer contributes any capacity. In contrast, the silicon in RNC@Si-50% still maintains its electrochemical reaction activity.

GCD profiles at 0.1 A g^−1^ in Figure 4d,e demonstrate the enhanced reversibility of RNC@Si-50%. It retains 74.0% of its initial capacity (1107.0 mAh g^−1^) after 10 cycles, compared with bare silicon with 67.2%. Fitting analysis of the electrochemical impedance spectroscopy (EIS) data (Figure 4f and and Figure S3 and Table S1 of the Supporting Information) shows that the introduction of the unique nodule-like structure reduces both the charge transfer resistance (Rct) and Warburg impedance (W_o_) of the RNC@Si composite. In the RNC@Si system, RNC acts as a fibrous carbon skeleton and forms a robust electronic conduction network—critical for facilitating charge transport. Since charge transfer resistance is directly correlated with a material’s electronic conductivity (higher conductivity translates to lower Rct), RNC@Si-10% (with a higher carbon skeleton content) exhibits enhanced electronic conductivity, resulting in a lower charge transfer resistance. Meanwhile, the abundant 3D porous structure of RNC can effectively facilitate the wetting and transport of the electrolyte, thereby endowing the RNC@Si composite with a lower W_o_ and significantly enhancing the lithium–ion diffusion rate. The rate capability test in Figure 4g highlights structural advantages. The RNC@Si-50% delivers a reversible capacity of 340.0 mAh g^−1^ at 0.5 A g^−1^, which is nearly tenfold higher than pristine silicon with only 32.5 mAh g^−1^. Long-term cycling in Figure 4h reveals capacity retention that correlates inversely with silicon loading. The RNC@Si-50% maintains a reversible capacity of 450.0 mAh g^−1^ after 400 cycles with an initial capacity retention of 40.6% compared with the RNC@Si-10% with 345.0 mAh g^−1^ and 57.0% retention. Despite a high initial capacity of 1757.0 mAh g^−1^ for pristine Si, only 8.9% of the initial capacity can be maintained after 400 cycles. During the charge–discharge process, the capacity fading of the silicon–carbon anode is mainly caused by the volume expansion and contraction of silicon. Therefore, the higher the mass proportion of silicon, the worse the cycling performance; consequently, the cycling stability of RNC@Si-10% is superior to that of RNC@Si-50%.

The coulombic efficiency curves (Figure S4 in Supporting Information) also indicate that RNC@Si exhibits superior electrochemical cycling stability. To evaluate the morphology of cycled electrodes, SEM images of RNC@Si-50% and pristine silicon electrodes after 300 cycles were compared. As shown in Figure 4i and Figure S5 in Supporting Information, the RNC@Si-50% electrode remains crack-free despite the volume fluctuations of Si. This resilience originates from the root-nodule design in which carbon shells constrain silicon expansion and fibrous networks dissipate stress. Although the RNC@Si-50% has certain advantages in electrochemical performance, further in-depth research is still needed to further enhance its performance.

## 3. Experimental Section

### 3.1. Materials

Silicon nanoparticles with an average particle size of ~100 nm were purchased from BTR New Material Group. Dimethyl sulfoxide (DMSO) and potassium hydroxide (KOH) were purchased from Sinopharm Chemical Reagent Co., Ltd. (Shanghai, China). Dopamine was purchased from Aladdin Biochemical Technology Co., Ltd. (Shanghai, China). Kevlar fiber was purchased from Blue Star New Chemical Material Co., Ltd. (Chengdu, China). The electrolyte LX-025 was purchased from DodoChem Reagent Co., Ltd. (Shenzhen, China).

Preparation of Aramid Nanofiber Solution: A homogenous KOH/DMSO solution was prepared by dissolving 1 g of KOH and 10 mL of deionized (DI) water in 450 mL of DMSO under constant stirring. Subsequently, 1.5 g of Kevlar fiber was introduced into the KOH/DMSO solution and stirred at 25 °C for 24 h to yield aramid nanofibers (ANFs) dispersion with a concentration of ~3.3 g L^−1^.

Synthesis of ANFs@Si-X and root-nodule carbon (RNC)@Si-X: Firstly, three groups of silicon nanoparticles (0.05, 0.15, and 0.25 g) were individually dispersed in 50 mL of DMSO. To promote surface functionalization, 0.05 g of dopamine was added to the solution and stirred at 40 °C for 2 h. The resulting solution was then dripped into 150 mL of ANFs solution and continuously stirred for 24 h to facilitate interfacial self-assembly. After self-assembly, 200 mL of DI water was slowly dripped into the mixture and stirred for 1 h to stabilize the composite structure. The resulting dispersion was centrifuged at 10,000 rpm for 5 min per cycle and repeatedly washed with DI water until a neutral pH was achieved. The final products of ANFs@Si-X composites were collected and freeze-dried, in which X stands for the different masses of silicon as starting materials (X = 10%, 30%, and 50%). The ANFs@Si-X precursors were pyrolyzed at 1000 °C with a heating rate of 5 °C min^−1^ for 3 h to yield RNC@Si-X (X = 10%, 30%, and 50%).

### 3.2. Materials Characterizations

Fourier-transform infrared spectroscopy (FTIR, Bruker, Vectory-80, Walzbach, Germany) was performed in the wavelength range of 800–3500 cm^−1^ to analyze chemical functional groups of samples. Surface area and pore size distribution of samples were examined through nitrogen adsorption–desorption isotherms recorded at 77 K using a JW-BK200C analyzer (JWGB, Bei Jing, China). X-ray diffraction (XRD, Nalytical, X’Pert PRO, Almelo, Netherlands) with Cu Kα radiation (λ = 0.15406 nm) was conducted at a scanning rate of 10° min^−1^ to identify the crystal phases of samples. X-ray photoelectron spectroscopy (XPS, Kratos AXIS Supra, Manchester, UK) was employed to investigate surface chemical states of samples. Morphological and microstructural features of samples were characterized using scanning electron microscopy (SEM, Hitachi, S-4800, Tokyo, Japan) and transmission electron microscopy (TEM, JEOL JEM-2010, Tokyo, Japan).

### 3.3. Electrochemical Measurements

Electrochemical measurements of samples were performed using 2032 coin cells assembled within an argon-filled (both O_2_ and H_2_O levels < 0.1 ppm) glove box. The working electrode was prepared by blending the active materials (pristine Si, RNC@Si-10%, RNC@Si-30%, and RNC@Si-50%), super P, carboxymethylcellulose (CMC), and styrene butadiene rubber dispersion (SBR, 48%) binder in a weight ratio of 80:10:5:5 in the presence of DI water as blending solvent. The slurry was coated onto Cu foil with an active material of ~1.0 mg cm^−2^. The cells were assembled with lithium metal foil as the counter/reference electrode, LX-025 as the electrolyte, and Celgard 2300 as the separator. Galvanostatic charge–discharge (GCD) measurements were conducted across a voltage range of 0.001–1.5 V vs. Li^+^/Li using a Land battery tester (Wuhan, China). Cyclic voltammetry (CV) and electrochemical impedance spectroscopy (EIS) were performed on a CHI 660E working station (Shanghai Chenhua Instrument Co., Ltd. (Shanghai, China)).

## 4. Conclusions

In summary, we successfully engineered a bioinspired Si@C anode material through a rational synthesis strategy involving PDA-mediated self-assembly of nano–silicon with aromatic polyamide nanofibers and subsequent controlled pyrolysis under Ar/H_2_ at 800 °C. The resulting hierarchical architecture, inspired by the root nodule symbiosis of leguminous plants, features vascular bundle-like carbon frameworks, nodule-like Si@C, and chemically bonded Si/C interfaces. The optimized composite delivers a high initial capacity of 1107.0 mAh g^−1^ at 0.1 A g^−1^ and maintains a stable capacity of 580.0 mAh g^−1^ after 100 cycles. The electrochemical performance is attributed to synergistic structural and interfacial engineering: the biomimetic fibrous carbon networks significantly reduce ionic diffusion impedance via 3D interconnected channels, while the biomimetic nodule-like Si@C enhances Li^+^ adsorption energy, and the graphitic carbon shell derived from polyamide decomposition effectively accommodates volume fluctuations and suppresses SEI degradation. This work highlights the potential of bioinspired design principles and multifunctional interfacial engineering in addressing critical challenges for high-capacity Si-based anodes, paving the way for advanced energy storage systems with long-term cycling stability.

## Data Availability

The original contributions presented in this study are included in the article/Supplementary Material. Further inquiries can be directed to the corresponding author(s).

## Supplementary Materials

The following supporting information can be downloaded at: https://www.mdpi.com/article/10.3390/molecules30214157/s1, Figure S1: The HRTEM image of Silicon; Figure S2: The dQ/dV curves of Silicon and RNC@Si-50% at (a) Cycle 1, and (b) Cycle 200; Figure S3: Complex-plane impedance fitted curves (a) Silicon, (b) RNC@Si-10%, (c) RNC@Si-30%, and (d) RNC@Si-50%; Figure S4: The coulombic efficiency of Silicon, RNC@Si-10%, RNC@Si-30%, and RNC@Si-50%; Table S1: Complex-plane impedance fitted data of Silicon, RNC@Si-10%, RNC@Si-30%, and RNC@Si-50%.
