# Supplementary material for: Silicon@Carbon Composite with Bioinspired Root-Nodule Nanostructures as Anode for High-Performance Lithium-Ion Batteries
Source: Molecules. 2025 Oct 22;30(21):4157. doi: 10.3390/molecules30214157 (PMC12608934; doi:10.3390/molecules30214157)
Supplement: Supplementary file 1 [file molecules-30-04157-s001.zip › molecules-3845278-supplementary.pdf]

Supporting Information for

Silicon@carbon Composite with Bioinspired Root-nodule Nanostructures as Anode for High-performance Lithium-ion Batteries

Yitong Sun[a,b*], Lei Zhao[a,b*], Ning Mi[a], Jiahao He[a], Jiantie Xu[c*]

[a] School of Materials Engineering, Longdong University, Qingyang 745000, Gansu, P. R. China

[b] Experimental Teaching Center of Mechanical Engineering, School of Intelligent Manufacturing, Longdong University, Qingyang 745000, Gansu, P. R. China

[c] School of Physics and Optoelectronics, South China University of Technology, Guangzhou 510640, P. R. China

* *Corresponding author. E-mail: Sunyitong0727@sina.com; zhaoleicrezy@163.com; jiantiexu@scut.edu.cn*

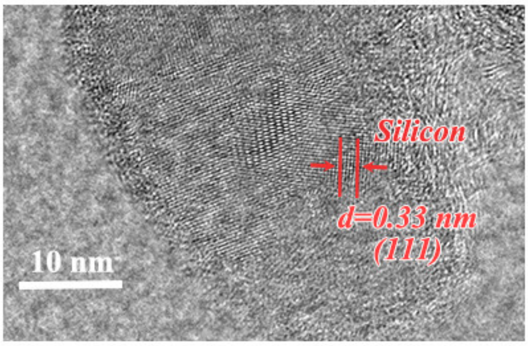

Figure S1. *The HRTEM image of Silicon.*

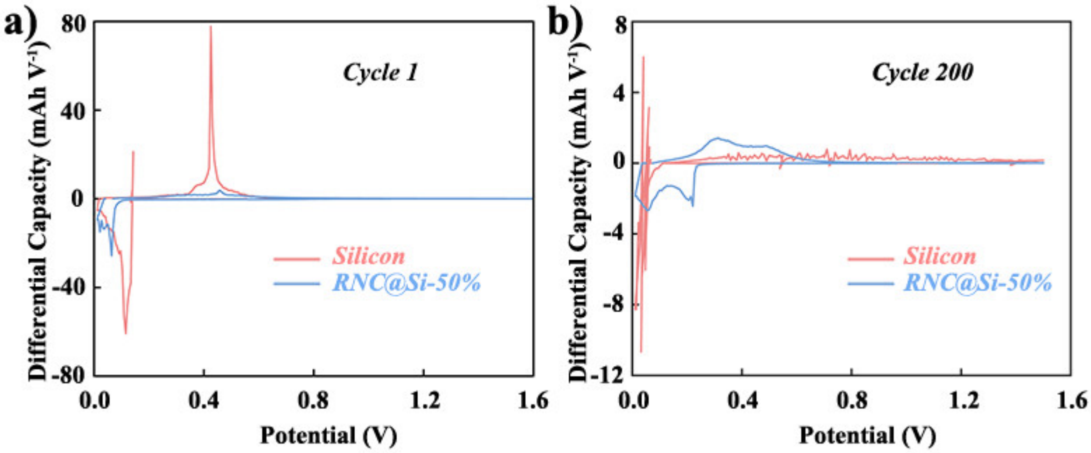

Figure S2. *The dQ/dV curves of Silicon and RNC@Si-50% at a) Cycle 1, and b) Cycle 200.*

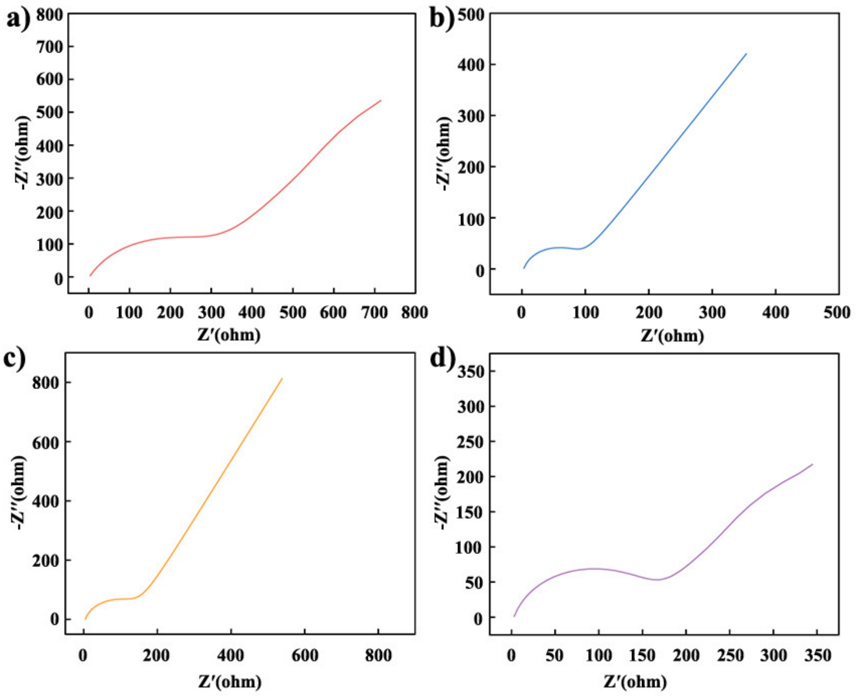

Figure S3. *Complex-plane impedance fitted curves a) Silicon, b) RNC@Si-10%, c) RNC@Si-30%, and d) RNC@Si-50%.*

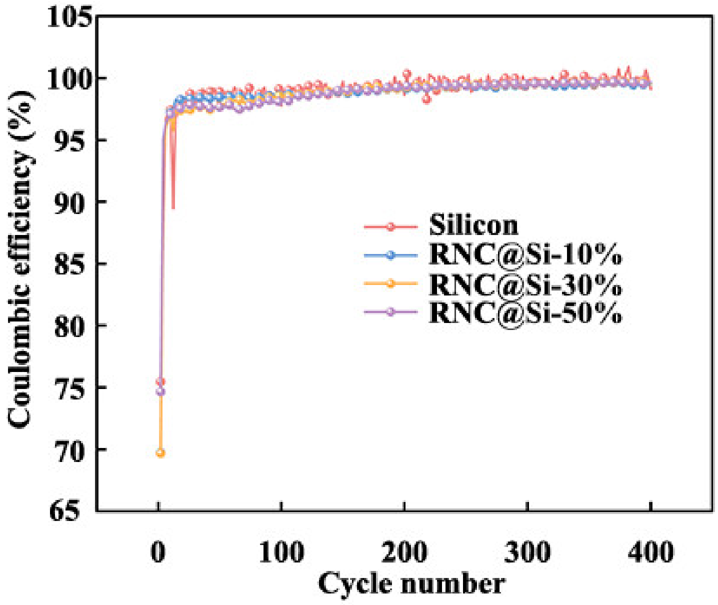

Figure S4. *The coulombic efficiency of Silicon, RNC@Si-10%, RNC@Si-30%, and RNC@Si-50%.*

Table S1. *Complex-plane impedance fitted data of Silicon, RNC@Si-10%, RNC@Si-30%, and RNC@Si-50%.*

Sample	R_s	R_{ct}	W_o
Silicon	1.8	290.1	0.58
RNC@Si-10%	2.9	82.3	0.31
RNC@Si-30%	4.0	129.2	0.35
RNC@Si-50%	2.6	150.8	0.48

The impedance data are calculated from the fitting curves as shown in ***Figure S3****.*
